# Diagnostic and Treatment Practices of Equine Endometritis—A Questionnaire

**DOI:** 10.3389/fvets.2020.00547

**Published:** 2020-09-02

**Authors:** Martin Köhne, Meike Kuhlmann, Anna Tönißen, Gunilla Martinsson, Harald Sieme

**Affiliations:** ^1^Clinic for Horses – Unit for Reproductive Medicine, University of Veterinary Medicine Hannover, Foundation, Hanover, Germany; ^2^Lower Saxony State Stud, Celle, Germany

**Keywords:** mare, inflammation, uterus, antibiotic resistance, survey, uterine lavage

## Abstract

Endometritis is a major cause for impaired fertility in mares. The objectives of this study were to collect information on diagnostic and treatment practices performed by veterinarians and to investigate possible effects of professional experience, caseload, and geographical location on the practitioners' management of endometritis cases. For this purpose, equine practitioners (*n* = 680) were asked to fill out an online survey (34 questions). The online survey yielded 117 responses by veterinarians practicing in all parts of Germany. Most respondents came from Lower Saxony and managed <20 mares per year. For the diagnosis of chronic infectious endometritis, uterine sampling for microbiological examination was performed manually with a swab by the majority of practitioners whereas only few used the speculum technique. The incidence of antibiotic resistant pathogens was reported to be <5% by almost all respondents. Most practitioners relied on systemic antibiotic treatment with trimethoprim-sulfadiazine. Only occasionally veterinarians used intrauterine antibiotic therapy. Uterine lavages were performed routinely by almost half of the respondents in case of positive uterine cultures, mostly with 0.9% saline solution. Irritant solutions (e.g., iodine, chlorhexidine, kerosene) were used less often. Collection of an endometrial culture after completion of the treatment was common practice. While only a very limited association of the geographical location of practitioner (e.g., on selection of mares for endometrial culture, *p* < 0.05) was observed, the number of managed mares affected the answers notably (e.g., for use of irritating intrauterine treatment, *p* < 0.05). The management of persistent breeding induced endometritis (PBIE) was influenced by the number of managed mares (e.g., for use of oxytocin, *p* < 0.05) and 29.6% of respondents administered antibiotics as part of their PBIE management. In summary, treatment strategies in the field vary considerably and include also non-evidence-based methods, but most German practitioners apply the recommended suitable treatments. Following the guidelines for antibiotic usage, most veterinarians administer antibiotics dependent on endometrial culture results but do not use cytology, low-volume lavage, or biopsy routinely. Antibiotic resistant uterine pathogens are reported to be isolated infrequently and equine practitioners prefer systemic to local antibiotic treatment of endometritis.

## Introduction

Endometritis in mares is among the major problems faced by equine practitioners (1). It is one of the main causes for impaired fertility in mares; chronic infectious endometritis (CIE) being observed in up to 25–60% of barren mares and resulting in reduced pregnancy rates and economic losses (2–4). Endometritis is defined as an inflammation of the endometrium and may be the result of several etiologies. An acute inflammatory response of the endometrium, which is characterized by the influx of polymorphonuclear neutrophils (PMNs), occurs physiologically after mating or insemination (5). Whereas, the inflammation passes within 48 h in normal mares, it persists in mares susceptible to endometritis (6). This condition is termed as persistent breeding-induced endometritis (PBIE) (7). In contrast to PBIE, which is caused by spermatozoa and other components of the ejaculate/insemination dose, CIE is characterized by the presence of microorganisms, most frequently *S. zooepidemicus* and *E. coli*, in the uterus (8, 9). Its occurrence is neither dependent on the cycle stage nor the time of mating. The clinical signs of CIE may be very subtle and microorganisms may not be found in every case. For such cases the term subclinical endometritis is used accordingly (4, 10). The present study focusses on CIE as well as PBIE. Other diseases of the equine uterus, e.g., endometriosis, pyometra, metritis, and contagious equine metritis (CEM), are not part of this investigation.

Several diagnostic methods have been described for the examination of endometritis. They can be divided into two steps: clinical examination including transrectal palpation and ultrasonography on the one hand, and sampling of the uterus via biopsy, swab, cytobrush, or low-volume lavage on the other hand (10). Whereas, an accumulation of intrauterine fluid (IUF) can be detected reliably via ultrasonography, thus enabling the veterinarian to diagnose acute endometritis (11), the underlying cause for IUF cannot be determined with certainty hereby. To this end, sampling techniques in conjunction with bacteriological, cytological and histological examinations are applied (10). Since each diagnostic tool has advantages and disadvantages, all of them are performed in practice (12).

Traditionally, CIE as well as PBIE treatment involves uterine lavages, administration of immunomodulatory agents and ecbolics such as oxytocin (4, 13). Although the usage of non-irritant solutions like saline [0.9% sodium chloride (NaCl) solution] or lactated Ringer's solution (LRS) is promoted extensively (14, 15), irritant solutions, e.g., povidone iodide solution (13, 16), or kerosene (17, 18), are used as well. In case of diagnosed CIE, antimicrobials are applied additionally. They are administered either systemically or via intrauterine instillation. Since there is conflicting evidence in the literature, both routes are used and may have specific advantages and disadvantages (12). However, the usage of antimicrobials should be performed reasonably and based on microbiological test results (12). Moreover, the instillation of mucolytics [e.g., dimethyl sulfoxide (DMSO) and N-acetylcysteine] for intrauterine treatment of bacterial biofilms has been promoted (12). Successful treatment of PBIE and CIE results in pregnancy rates as high as in reproductively healthy mares according to some authors (19), whereas others described reduced pregnancy rates in mares, diagnosed, and treated for endometritis (20, 21). Notwithstanding, endometritis remains one of the major reasons for impaired fertility if left untreated (3, 4).

Since no studies on the diagnostic and treatment procedures performed by German equine practitioners yet exist, this study aims to provide information on how veterinarians diagnose and treat endometritis in mares. Furthermore, possible differences regarding the handling of endometritis are hypothesized among practitioners according to their number of managed mares per year, their professional experience and their geographical location in Germany. For this purpose, an online-survey was performed among veterinarians with a reported interest in equine medicine.

## Methods

### Study Population and Survey Delivery

The study population consisted of veterinarians with a reported interest in equine medicine. Mailing lists used for advertisement of advanced training courses by the Clinic for Horses, University of Veterinary Medicine Hannover, Germany, as well as online search for equine practitioners were used to collect e-mail addresses of possible participants. In total, 680 veterinarians were contacted via e-mail and asked for their participation in the online survey. The respondents did not need a registration but had to follow the link to the questionnaire. The online survey was created and performed using the LimeSurvey platform (www.limesurvey.org; LimeSurvey GmbH, Hamburg, Germany). The survey was conducted over an 8-week period in 2019 (April–June) and participation was voluntary.

### Questionnaire

The questionnaire consisted of 34 questions, divided into five subsets: General data on the practitioner, diagnostic procedures for endometritis, management of CIE, management of PBIE, and abandoned diagnostic and therapy methods. Questions were either designed as single or multiple choice questions. Comment boxes were offered whenever appropriate. Some questions were coupled so that certain questions only appeared if a certain answer had been given before (e.g., question B was coupled to answer 1 of question A but not to answer 2 of question A). An English version of the questionnaire has been added as Supplementary Material.

### Ethics

The survey was reviewed by the data protection officer of the University of Veterinary Medicine, Hannover, Germany and approved as legal according to European and German General Data Protection Regulations. All participants in the survey remained anonymous and gave their formal consent for publication of the results. The study was approved as ethical by an institutional review board (Doctoral Commission, Stiftung Tierärztliche Hochschule Hannover, 2019, 3.5).

### Analysis

Raw data were provided as an Excel sheet (Microsoft Germany GmbH, Unterschleißheim, Germany) by the LimeSurvey platform. Data were analyzed using IBM SPSS software (SPSS 26, SPSS, Armonk, New York, USA). After descriptive analysis of the data, groups of respondents were formed according to respondents' answers for number of managed mares (<20; 21–40; 41–70; 71–100; 101–150; 151–200; >200) and geographical location in Germany (Lower Saxony = Lower Saxony; North = Mecklenburg-Western Pomerania, Schleswig-Holstein; East = Brandenburg, Saxony, Saxony-Anhalt; Central = Hesse, Rhineland-Palatinate, Saarland, Thuringia; South = Baden-Wuerttemberg, Bavaria; West = North Rhine-Westphalia). Using Pearson's *X*^2^-test and Fisher's Exact test, answers for diagnostic procedures and treatment methods for endometritis as well as management of PBIE were compared among groups. Significance was determined at *p* < 0.05.

## Results

### Survey Response

A total of 117 respondents partially or fully completed the survey, resulting in a return rate of 17.2%. Since not every question was relevant to every respondent and not every survey was filled out entirely, the number of respondents for each question varied. Nearly half of the veterinarians practice in Lower Saxony (44.3%; 52/116). Less than 10% (8.8%; 10/114) manage more than 200 broodmares per year. The majority of respondents had more than 10 years of professional experience (75%; 87/116). More detailed information on the participants in the survey is given in Table 1.

**Table 1:** General information on participants in the survey.

Years of professional experience		1–2 years	3–5 years	6–10 years	11–20 years	21–30 years	>30 years
Total number (*n* = 116)		5	7	17	43	29	15
Geographical region of practice in Germany		Lower Saxony	North	East	Central	South	West
Total number (*n* = 116)		52	11	16	10	17	10
Number of managed mares per year	<20	21-40	41–70	71–100	101–150	151–200	>200
Total number (*n* = 114)	33	23	20	13	10	5	10

*Years of professional experience, geographical region of practice in Germany and number of managed mares per year as reported by the participants in the survey. Geographical region of practice: Lower Saxony = Lower Saxony; North = Mecklenburg-Western Pomerania, Schleswig-Holstein; East = Brandenburg, Saxony, Saxony-Anhalt; Central = Hesse, Rhineland-Palatinate, Saarland, Thuringia; South = Baden-Wuerttemberg, Bavaria; West = North Rhine-Westphalia*.

### Applied Diagnostic Methods for Endometritis

**Figure 1:**
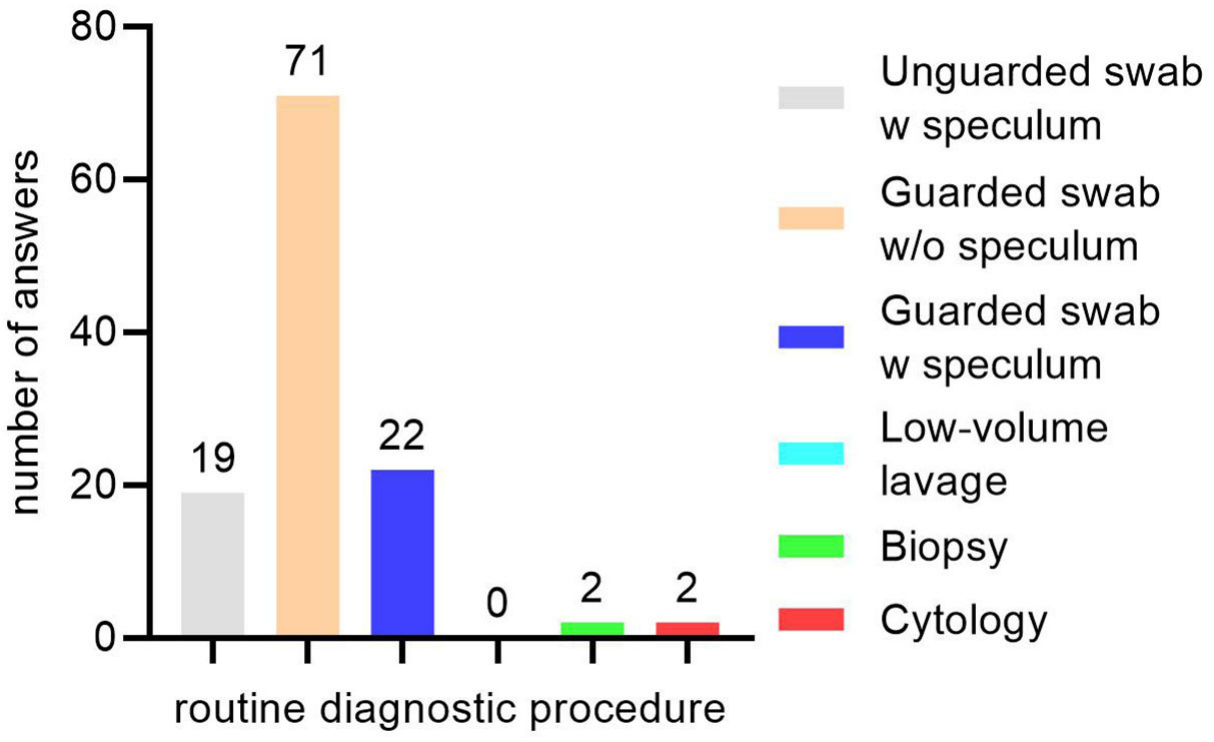
Routine diagnostic procedures for equine endometritis performed by practitioners in Germany. Number above columns indicate the number of answers for the specific diagnostic tool. “w,” with; “w/o,” without.

**Figure 2:**
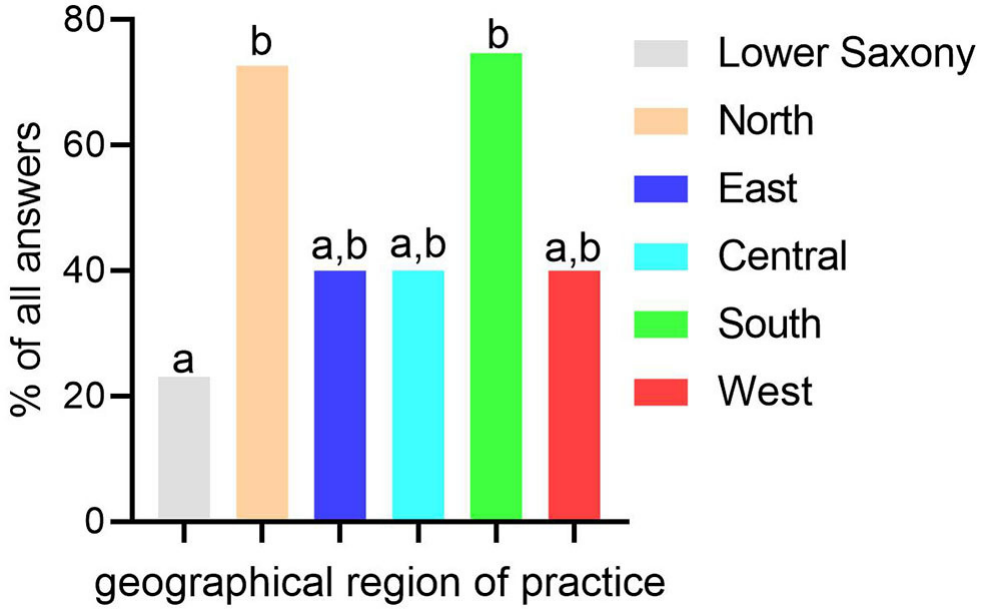
Routine uterine sampling of young maiden mares (<5 years old) for microbiological examination in relation to geographical region of practice in Germany. Geographical region of practice: Lower Saxony = Lower Saxony; North = Mecklenburg-Western Pomerania, Schleswig-Holstein; East = Brandenburg, Saxony, Saxony-Anhalt; Central = Hesse, Rhineland-Palatinate, Saarland, Thuringia; South = Baden-Wuerttemberg, Bavaria; West = North Rhine-Westphalia. Columns with different superscripts (a,b) represent statistically significant difference among groups (*p* < 0.05).

In Germany, the most popular routine sampling technique for diagnosis of endometritis is the use of a guarded swab that is manually introduced into the uterus (61.2%; 71/116). Cytology is performed routinely by 1.7% of practitioners (2/116; Figure 1). Regarding the selection of mares subjected to sampling techniques, geographical differences within Germany were observed. In Lower Saxony, practitioners perform uterine sampling in young maiden mares (3–4 years old) significantly less routinely (23.1%; 12/52) than practitioners from the North region (72.7%; 8/11; *p* < 0.01; Figure 2). The microbial species isolated from the uterus by the respondents may be ranked according to the frequency of isolation in the following order: ß-hemolytic *Streptococci* ssp., *E. coli*, α-hemolytic *Streptococci* ssp., *Enterococcus* ssp., *Pseudomonas* ssp., *Klebsiella* ssp., yeast and other fungi. Asked for multidrug-resistant bacteria within their isolates, ~90% of the practitioners reported an incidence of 0–5% (0% multidrug-resistant bacteria = 45.2%; 52/115, 1–5% = 44.4%; 51/115). Obtaining control samples for microbiological examination after treatment of CIE is common practice in Germany (87.9%; 102/116). None of the practitioners collects an endometrial culture earlier than 3–5 days after the end of treatment and 66.7% (44/66) obtain the control sample at least 9 days after the end of treatment. The remaining respondents sample the uterus either after 3–5 days (13.6%; 9/66) or after 6–8 days (19.7%; 13/66) following the last day of treatment. No influences of professional experience or number of mares managed per year were detected.

### Management of CIE

The majority of respondents uses systemic antibiotic administration for CIE treatment (77.7%; 87/112), whereas <20% of respondents perform intrauterine instillation of antibiotics (17.9%; 20/112), and <5% do not use any antibiotics for CIE treatment (4.5%; 5/112). If treating systemically, most practitioners administer antibiotics for at least 5 days (89.7%; 79/87) and the preferred antibiotic agent is trimethoprim-sulfadiazine (administered by 57.5%; 50/87), followed by procaine-penicillin G(31.0%; 27/87), and a combination of procaine-penicillin G and gentamicin (4.6%; 4/87). Ceftiofur and fluorchinolones are administered by 2.3% (2/87) each. If antibiotics are administered locally, most practitioners do not administer antibiotics for more than 3 days (36.8%; 7/19) and 36.8% (7/19) use amoxicillin pills registered for intrauterine application, whereas 63.2% (12/19) infuse antibiotics that are not registered for intrauterine use in horses in Germany (e.g., ceftiofur, gentamicin, and penicillin). No differences in the use of antimicrobials were observed between groups of practitioners from different geographic locations, with differing years of professional experience or according to numbers of mares managed.

Regarding uterine lavages, ~90% of practitioners use 0.9% saline solution (88.2%; 90/102). The use of irritant solutions like kerosene is reported as well (2.9%; 3/102; Table 2). Furthermore, many other substances and solutions are used for intrauterine infusion or lavage, e.g., hydrogen peroxide, eucalyptus oil, polyhexanide, policresulen (Lotagen®, Intervet, Unterschleißheim, Germany), LRS, plasma, tea tree oil, chloramine-T, and yogurt. Significant differences between groups of respondents exist for the use of 0.9% saline solution in mares. This solution is used more often by practitioners from Lower Saxony (95.7%; 45/47) and the West region (100%; 10/10) as compared to other regions (*p* < 0.05; Table 3). Furthermore, the number of managed mares has an influence on the usage of ethacridine lactate solution and tap water. Veterinarians who manage more than 70 mares employ both solutions more often (*p* < 0.05; Table 4). Only 10.3% of all respondents (12/117) do not administer oxytocin at all, whereas 89.7% (102/117) use it either routinely after uterine lavages or after detecting echogenic intrauterine fluid via transrectal ultrasound (Table 4). Pregnancy rate per cycle after treatment of CIE is reported to be ≤ 50% by the majority of practitioners (86.6%; 75/90), whereas the odds for pregnancy per year after treatment were estimated to exceed 70% by 45.1% of respondents (41/91). No effects of the practitioners' professional experience on CIE management were observed.

**Table 2:** Solutions used for uterine lavage/instillation by practitioners in Germany.

**Solution**	**Mentions (*n* = 188)**
0.9% NaCl solution	90
Tap water	6
Ethacridine lactate solution	19
Iodine solution	33
DMSO	2
Chlorhexidine	5
N-acetylcysteine	9
Coca Cola	10
Kerosene	3
Other	11

*Answers for “Other”: hydrogen peroxide, Euca-Comp (2x), ozone, polyhexanide, policresulen, Lactated Ringer solution, plasma, tea tree oil, chloramine T, yoghurt. DMSO, dimethyl sulfoxide*.

**Table 3:** Differences in usage of solutions for uterine lavage/instillation for treatment of chronic infectious (CIE) and persistent breeding induced endometritis (PBIE) by geographical region of practice in Germany.

**Geographical region of practice in Germany**	**Endometritis type**	**Lower Saxony**	**North**	**East**	**Central**	**South**	**West**	***X*^**2**^**
Use of 0.9% NaCl solution for uterine lavage	CIE	95.7% (45/47)	75.0% (6/8)	78.6% (11/14)	77.8% (7/9)	76.9% (10/13)	100% (10/10)	*p* = 0.04
	PBIE	70.0% (35/50)	45.5% (5/11)	37.5% (6/16)	80.0% (8/10)	47.1% (8/17)	80.0% (8/10)	*p* = 0.04

*Geographical region of practice: Lower Saxony = Lower Saxony; North = Mecklenburg-Western Pomerania, Schleswig-Holstein; East = Brandenburg, Saxony, Saxony-Anhalt; Central = Hesse, Rhineland-Palatinate, Saarland, Thuringia; South = Baden-Wuerttemberg, Bavaria; West = North Rhine-Westphalia*.

**Table 4:** Differences in usage of solutions for uterine lavage/instillation for treatment of chronic infectious (CIE) and persistent breeding induced endometritis (PBIE) by number of managed mares per year in Germany.

**Endometritis type**	**Number of managed mares per year**	**<20**	**21–40**	**41–70**	**71–100**	**101-150**	**151–200**	**>200**	***X*^**2**^**
CIE	Use of tap water for uterine lavage	0% (0/28)	5.6% (1/18)	6.3% (1/16)	23.1% (3/13)	0% (0/10)	20.0% (1/5)	0% (0/10)	*p* = 0.04
	Use of ethacridine lactate solution for uterine lavage	3.6% (1/28)	16.7% (3/18)	6.3% (1/16)	38.5% (5/13)	30.0% (3/10)	40.0% (2/5)	40.0% (4/10)	*p* = 0.01
PBIE	Use of oxytocin	37.5% (15/32)	60.9% (15/23)	47.4% (9/19)	69.2% (9/13)	70.0% (7/10)	100% (5/5)	100% (10/10)	*p* = 0.01
	Use of corticosteroids	0% (0/32)	17.4% (4/23)	31.6% (6/19)	38.5% (5/13)	30.0% (3/10)	60.0% (3/5)	40.0% (4/10)	*p* = 0.01
	Use of antibiotics	18.8% (6/32)	26.1% (6/23)	31.6% (6/19)	38.5% (5/13)	60.0% (6/10)	0% (0/5)	10.0% (1/10)	*p* = 0.02

### Management of PBIE

Asked for components of their PBIE management, 65.2% of the respondents (75/115) named induction of ovulation, 61.7% (71/115) perform uterine lavages with non-abrasive solutions (0.9% NaCl solution and tap water) and 59.1% (68/115) administer oxytocin. Non-steroidal anti-inflammatory drugs (NSAIDs; 17.4%; 20/115), corticosteroids (21.7%; 25/115) and antibiotics (29.6%; 34/115) are used by <50% of practitioners. However, management of PBIE is influenced by the geographical location of the practitioner and the number of mares managed. Practitioners that manage 151-200 mares per season use oxytocin (100%; 5/5; vs. 60.9%; 14/23; *p* < 0.05), corticosteroids (60.0%; 3/5; vs. 17.4%; 4/23), and uterine lavages with non-irritant solutions more often than practitioners managing 21–40 mares (100%; 5/5; vs. 65.2%; 15/23; *p* < 0.05; Table 4). Conversely, antibiotics are administered less frequently by veterinarians handling 151–200 mares (0%; 0/5) as compared to practitioners dealing with 71–100 mares per year (38.5%; 5/13; *p* < 0.05; Table 3). The location (region) of the practitioner influences the use of 0.9% saline as described for CIE treatment (*p* < 0.05; Table 3). The professional experience of the respondents did not affect their management of PBIE significantly.

### Abandoned Diagnostic and Therapy Methods

Participants in the survey had the opportunity to answer the questions “Which diagnostic/treatment methods did you give up?” via comment boxes. For abandoned diagnostic methods, 46 responses were given and summarized in Table 5. Approximately 25% (24.4%; 11/46) had not given up any diagnostic method, whereas the same number of participants had abandoned performing uterine biopsy (11/46). For abandoned therapy methods, 61 answers were received (Table 5). Around one third of respondents (35.6%; 23/61) named intrauterine antibiotics as an abandoned therapy method and 37.5% (26/61) do not use irritant solutions for uterine lavages any longer.

**Table 5:** Abandoned diagnostic and treatment methods for endometritis as reported by equine practitioners in Germany.

**Abandoned diagnostic methods**	**Answers (*n* = 46)**	**Abandoned therapies**	**Answers (*n* = 61)**
None	11	Intrauterine antibiotics	23
Endometrial biopsy	11	Antimicrobials	3
Cytology	8	Uterine lavage	4
Uterine endoscopy	3	Instillation of irritating solutions:	26
Vaginoscopy	4	Iodine	8
Uterine swabbing with speculum	5	Policresulen	5
Uterine swabbing without speculum	1	Coca Cola	2
Low-volume Lavage	1	Ethacridine lactate	3
Uterine swabbing at all	2	DMSO	1
		Kerosene	2

*DMSO, dimethyl sulfoxide*.

## Discussion

Endometritis is a problem that is frequently encountered by equine practitioners (1) and has been researched thoroughly during the last decades (12). However, information on how veterinarians in the field diagnose and treat endometritis is scarce. According to the knowledge of the authors, only two studies investigating the diagnostic and treatment practices of endometritis in France (22) and the USA (23) exist. In the context of stricter legal regulations regarding the use of antimicrobial agents in animal husbandry and in order to change anecdotally performed treatment methods to evidence-based strategies, more investigations of this kind are required. The aim of this study was thus to collect and provide information regarding diagnostics and therapies of CIE and PBIE in Germany. Based on our survey results, differences in diagnostic and treatment practices among practitioners were determined. Additionally, treatment strategies involving non-evidence-based approaches as well as off-label use of drugs were reported by some practitioners. However, antibiotic treatment is performed responsibly by most veterinarians.

A large number of veterinarians from every part of Germany and with differing expertise in equine reproduction took part in the survey. Thus, a broad overview over the management of equine endometritis in Germany is provided. It is noteworthy that comparable surveys on issues of equine clinical practice such as retained fetal membranes (24), nasogastric intubation (25), or management of endometritis (22, 23) were completed by a comparable or smaller number of veterinarians. Since the exact number of practitioners working with equines is not gathered in Germany, no statement of the proportion of participants in the survey to the total number of equine practitioners is possible. Therefore, it is not the claim of this survey to be representative but to give a broad overview of the endometritis management in the field.

Given the fact that a vast number of research papers and reviews has been published on various methods for the diagnosis of equine endometritis over the years (10, 26–31), the results of the survey are unexpected. It appears that contrary to their broad acceptance in research cytology and low-volume lavage are not performed routinely in Germany, although cytology can be performed easily and quickly provides information on the presence of subclinical endometritis (27, 32). Furthermore, cytology is used by almost two thirds of practitioners in the USA in conjunction with uterine swabbing (23), yielding a more accurate diagnosis of endometritis. Performing a low-volume lavage is indeed more labor-intensive than uterine swabbing, but results are more accurate (28, 33). However, this technique is rather used as an addition to a primary diagnostic tool (12), which might explain the low number of mentions for this method in the present study. Nevertheless, practitioners in France are using low-volume lavage more frequently (22). Apparently, there is a knowledge gap of the advantage these two techniques by practitioners in Germany that should be closed by a stronger advertisement of these tools. A study from our group (31) clearly showed the benefits of the speculum technique for obtaining endometrial cultures, which led to a significant reduction of uterine contamination during the sampling process. However, due to the requirement of an additional person for holding the speculum, the practical feasibility of this technique is impaired in the field and therefore not predominantly used by practitioners in the field. Fortunately, double-guarded swabs for uterine microbiology sampling are widespread in Germany, leading to less contamination of the sample and subsequently to more adequate microbiological examination results (28). Surprisingly, the use of non-guarded swabs was reported by 16% of practitioners, illustrating the difficulty of eradicating antiquated practices.

Until several years ago, the dogma of uterine sterility, which was refuted recently (34–37), was taught by the universities. Despite the fact that uterine insterility was demonstrated (34–37), it is not necessary to take uterine samples for microbiological examination in young maiden mares (<5 years old), unless abnormalities have been reported or detected during the gynecological examination, according to the authors' opinion, This opinion is in contrast to the regulations for Thoroughbred mares (38, 39).

The main horse breeding regions are located in the Northwest, including Lower Saxony, North Rhine-Westphalia and Schleswig-Holstein. Since equine reproduction in other parts of Germany is not that important, less informed equine veterinarians may have different approaches (e.g., endometrial culture of young maiden mares).

Not surprisingly, endometrial biopsies are not used as a standard diagnostic tool for the detection of endometritis, probably due to its invasive nature and the availability of different less invasive sampling methods. Furthermore, they are rather used as additional as primary diagnostic tools. Participants in the survey even named the use of this technique as a method they had given up during their career. According to the literature however, endometrial biopsy sampling is still the gold standard for endometritis (28, 40) as well as endometriosis diagnostics (41) and should therefore be valued as an important additional diagnostic tool.

In accordance with other studies (4, 9, 28–30), ß-hemolytic *Streptococci* ssp. and *E. coli* had the highest incidence among uterine microbial isolates, followed by other bacteria species like *Pseudomonas* ssp*., Klebsiella* ssp*., Staphylococcus* ssp., and fungi. In contrast to study results from Italy (42), Slovakia (43), and Sweden (44), most respondents estimated the incidence of antibiotic resistant bacteria to be <5%. While the results of this study were obtained via questionnaire and thus only reflect an estimation by the respondents, the results of the mentioned studies from Italy, Slovakia and Sweden are based on actual laboratory data. This might explain the differing results. However, a more profound database on antibiotic resistance of uterine bacterial isolates is required to verify this hypothesis.

Acting in accordance with the guidelines published by the German Federal Chamber of Veterinarians (45) and the World Organization for Animal Health (OIE) (46), most practitioners obtain a control sample for microbiological examination routinely after the antimicrobial treatment of CIE. According to German literature, it is recommended to perform control swabbing (38) not earlier than 10 days post treatment (47, 48). Since almost two thirds follow this advice, the participants in the survey appear to be aware of this recommendation and the guidelines.

In contrast to reports from other countries, like France (22), the UK (49), and the USA (23, 50, 51), intrauterine use of antibiotics is uncommon among respondents in this study. They clearly prefer to administer antibiotics systemically for at least 5 days. As reviewed by Canisso et al. (12), there is still conflicting evidence concerning the most suitable route for administration of antimicrobials for endometritis treatment in mares. Although systemic treatment may result in severe complications such as diarrhea, colitis, anaphylactic reactions (23), and disruption of fecal microbiota (52), the effectiveness of systemic treatment seems to outweigh the possible complications in the practitioners' perspectives. Correspondingly, many respondents reported to have abandoned intrauterine use of antibiotics in favor of systemic administration, because it was perceived to result in a more favorable outcome. In fact, the efficacy of many antibiotics, e.g., trimethoprim sulfadiazine (53) and gentamicin (54), for the systemic treatment of equine endometritis has been demonstrated. Furthermore, reservations regarding the costs of systemic antibiotics and veterinary service (13) are unwarranted, since oral treatment with trimethoprim sulfadiazine is inexpensive and effective against a broad spectrum of bacteria (53). Due to these advantages, it is the antibiotic of choice for the majority of the participants in the study, although it severely reduces bacterial diversity in the fecal microbiota (52). In contrast to the decreasing usage of intrauterine antibiotics in Germany, the use of covering therapies (intrauterine antibiotics, uterine lavage and oxytocin treatment) has increased from 12.21% in 1998 to 62.7% in 2013/2014 in Thoroughbred mares in the UK (55, 56). Strikingly, 49.6% of Thoroughbred mares received intrauterine antibiotics in 2013/2014, although Allen et al. demonstrated similar pregnancy rates per cycle on day 15 after ovulation (regardless of the fact whether antibiotics were infused into the uterus or uterine lavage with 0.9% NaCl solution in combination with oxytocin administration was performed) (57). As already stated by Rose et al. (56), the high use of covering therapies in Thoroughbreds in the UK may be questioned.

Regarding intrauterine use of antimicrobials, solely amoxicillin pills are registered in Germany for this route of administration. However, these products appear to be unpopular among practitioners for endometritis treatment in mares, since more respondents rely on the local use of other antimicrobial agents (e.g., ceftiofur, gentamicin, and penicillin), the use of which has been reviewed extensively by other authors (13, 51). Despite their clinical efficacy, none of these agents are registered for intrauterine treatment of equine endometritis in Germany. According to the prescribing cascade, the use of these antibiotics is difficult to justify due to the availability of amoxicillin pills for intrauterine and other antibiotics for systemic treatment (e.g., trimethoprim sulfadiazine) of the same condition. In the future, this practice may get into the focus of control authorities. Additionally, it should be emphasized that a microbiological examination and susceptibility testing should be performed prior to any antibiotic treatment (45, 46, 51) and a suitable antibiotic should be chosen according to the test results, which is done by most of the practitioners in accordance with the guidelines. However, the use of antibiotics for treatment of PBIE as reported by almost one third of veterinarians is opposing recommended treatment strategy since bacteria are usually not involved crucially in PBIE (15, 58).

Although the use of 0.9% saline is common practice among respondents of this study and practitioners worldwide (14), instillation of more irritant solutions such as iodine is performed concurrently. Participants in the study managing more than 70 mares also reported lavage with ethacridine lactate. As for many other substances, there is no evidence for their safety and efficacy for intrauterine application or for their effects on the resident uterine microbiome (12). The use of tap water for uterine lavages might be attributed to its ubiquitous availability at a high hygienic quality and its low cost. Further research is required to provide practitioners with more evidence-based, non-antibiotic treatment options for CIE. Notwithstanding, many participants in the survey stopped using irritant solutions during their career. Therefore, we conclude that uterine lavages with 0.9% saline are sufficient in most CIE and PBIE cases, whereas only some cases may require treatment with different solutions, e.g., povidone iodine, N-acetylcysteine, hydrogen peroxide, dimethyl sulfoxide (DMSO), or kerosene (12, 59, 60).

According to the literature, endometritis treatment should include oxytocin administration (61–63). Most of the participants in the survey act in accordance with these recommendations and use oxytocin routinely, after uterine lavages or if intrauterine fluid has been detected ultrasonographically.

Besides the use of ecbolics and uterine lavages, management strategies of PBIE should incorporate several other elements to improve treatment success (15). A single post-breeding antibiotic treatment is not evidence-based (56). Uterine lavages and ecbolics are the recommended treatments (64). In our survey, the majority of practitioners included uterine lavages with 0.9% saline solution, oxytocin and induction of ovulation into their management of PBIE. When breeding or inseminating a mare susceptible to PBIE, only one insemination or mating per estrus is of advantage in order to decrease the inflammatory stimulus (5). This can be more easily achieved when ovulation is induced (65). Veterinarians managing >200 mares applied the management of PBIE (no antibiotics, oxytocin, and uterine lavage with 0.9% saline solution). Thus, these practitioners apply the recommended treatments best, probably due to the importance of equine reproduction in their daily clinical work and a resulting interest in new developments in the clinical research. The use of immunomodulators to manage susceptible mares is not as widespread among the participants as the aforementioned management strategies. As reported by multiple authors, the use of glucocorticoids (66, 67) as well as NSAIDs (68) is beneficial for improving pregnancy rates in PBIE affected mares. Apparently, practitioners managing more mares and those being located in the main breeding regions in Germany are more familiar with these treatment approaches.

In general, the need for a better knowledge transfer from clinical research to practitioners became obvious in this study. According to our knowledge, many therapy methods performed by practitioners (e.g., uterine lavage with policresulen) have not been taught at the universities for the past 30 years. Therefore, it remains unclear how these outdated methods survived in the field. Again, continuing education of veterinarians seems to have a pivotal role here and the influence of senior veterinarians on young graduates regarding their choice of diagnostic and treatment procedures for endometritis has to be investigated. One study from Belgium showed, that practitioners rather relied on consultation of colleagues, specialists, laboratories, and the internet than scientific data bases and peer-reviewed journals in a decision-making process (69). However, more research on the practitioners' motives for their chosen diagnostic and therapeutical procedures is needed.

## Conclusion

In conclusion, most practitioners perform uterine endometrial swabbing as a routine diagnostic practice for endometritis, but endometrial cytology is used very rarely and low-volume lavage not at all as a routine diagnostic procedures. Some old non-evidence based treatment approaches are still widely used, but most veterinarians managing over 150 mares apply treatment practices that are in line with current recommendations in the literature. To update the diagnostic and therapeutic practices of equine practitioners and to promote evidence-based treatments, a more effective and targeted dissemination of the scientific knowledge to veterinarians and more clinical research on equine endometritis therapies is necessary. Moreover, a mostly cautious and reasonable use of antibiotics for endometritis treatment was reported in this study. This practice should be maintained and supported to conserve or even improve the situation of antibiotic resistance of bacterial endometritis pathogens.

## Data Availability Statement

The raw data supporting the conclusions of this article will be made available by the authors, without undue reservation.

## Ethics Statement

The survey was reviewed by the data protection officer of the University of Veterinary Medicine, Hannover, Germany and approved as legal according to European and German General Data Protection Regulations. All participants in the survey remained anonymous and gave their formal consent for publication of the results after having read and accepted the conditions of participation in the survey by clicking the “Accept”-button on the online survey homepage. This procedure was in line with Art. 360 I 1 lit. a GDPR (German General Data Protection Regulations). This study was approved as ethical by an institutional review board (Doctoral Commission, Stiftung Tierärztliche Hochschule Hannover, 2019, 3.5).

## Author Contributions

MKö, MKu, AT, GM, and HS participated in the design of the study. MKö and MKu collected the data and performed the statistical analyses. MKö and HS drafted the manuscript. All authors read and approved the final manuscript.

## Conflict of Interest

The authors declare that the research was conducted in the absence of any commercial or financial relationships that could be construed as a potential conflict of interest.
