# Supplementary material for: Diagnostic and Treatment Practices of Equine Endometritis—A Questionnaire
Source: Front Vet Sci. 2020 Sep 2;7:547. doi: 10.3389/fvets.2020.00547 (PMC7492380; doi:10.3389/fvets.2020.00547)
Supplement: Supplementary file 1 [file Data_Sheet_1.docx]

Questionnaire: Diagnosis and Treatment of Endometritis in Mares in Germany

SC: single-choice question

MC: multiple-choice question

1. General Data
   1. For how long are working as a veterinary practitioner? (SC)
      - 1-2 years
      - 3-5 years
      - 6-10 years
      - 11-20 years
      - 21-30 years
      - More than 30 years
   2. Indicate the State Chamber of Veterinarians to which you belong? (SC)
      - Baden-Wuerttemberg
      - Bavaria
      - Berlin
      - Brandenburg
      - Hesse
      - Mecklenburg- Western Pomerania
      - Lower Saxony
      - North Rhine
      - Rhineland-Palatinate
      - Saarland
      - Saxony-Anhalt
      - Thuringia
      - Westphalia-Lippe
   3. What is the type of practice, you are working for? (SC)
      - Single practice
      - Mixed practice
      - Clinic
      - Stud farm
      - Other
        - comment box
   4. What is the proportion of equine reproductive medicine in your practical work? (SC)
      - Less than 10%
      - 11-20 %
      - 21-30 %
      - 31-40 %
      - More than 40 %
   5. Which services for equine reproductive medicine do you offer? (MC)
      - Management of natural mating
      - Artificial insemination
      - Embryo transfer
      - Ovum pick-up
      - Stud farm practice
   6. How many mares are you managing per year? (SC)
      - Less than 20
      - 21-40
      - 41-70
      - 71-100
      - 101-150
      - 151-200
      - More than 200
2. Diagnostic Procedures for Endometritis
   1. Which form of endometritis do you encounter most of the times? (SC)
      - Chronic infectious endometritis (CIE)
      - Venereally transmitted endometritis (e.g. CEM)
      - Persistent-breeding induced endometritis (PBIE)
      - Other
        - Comment box
   2. Do you take uterine samples for microbiological examination? (SC)
      - Yes
      - No
        - If 2.b. was “No”:
          - How do you determine the form of endometritis alternatively?

Comment box

- - - - If 2.b. was “Yes”:
        - Which technique do you use routinely for bacteriological sampling of the uterus? (SC)

Swab with speculum

Double guarded swab without speculum

Double guarded swab with speculum

Low-volume lavage

Biopsy

Cytology

- - - - - What is your preferred cycle stage for uterine sampling? (SC)

Anestrus

Diestrus

Estrus

No preference

- - - - - In which mares do you perform uterine sampling routinely? (MC)

Young maiden mares (3-4 years old)

Older maiden mares (older than 4 years of age)

Foaling mares

Barren mares

Slipped mares

Mares after abortion/resorption

Mares with a history of dystocia

Other:

Comment box

- - - - - Rank the infectious agents according to their incidence within your uterine samples:

α-hemolytic Streptococci

ß-hemolytic Streptococci

E. coli

Pseudomonas ssp.

Klebsiella ssp.

Enterococcus ssp.

Yeast (e.g. Candida ssp.)

Mould (e.g. Aspergillus ssp.)

- 1. What is the percentage of multi-resistent bacteria in microbiological samples obtained from your patient material?

0%

1-5%

6-10%

11-20%

More than 20%

- 1. Do you perform endometrial biopsies as an additional diagnostic tool for endometritis? (SC)
     - Yes
     - No

1. Management of CIE
   1. How do you treat mares with a positive uterine culture routinely? (SC)
      - Systemic antibiotic treatment
      - Intrauterine antibiotic treatment
      - Without antibiotics
        - If 3.a. was “Systemic antibiotic treatment”:
          - Which antibiotic drug / combination of antibiotic drugs do you use most of the times? (SC)

Trimethoprim-sulfadiazine

Procaine penicllin G

Gentamicin

Procaine penicillin G / Gentamicin

Ceftiofur

Other:

Comment box

- - - - - How long do you treat mares with a positive uterine culture on average? (SC)

One day

2 days

3 days

4 days

5 days

6 days

7 days

More than 7 days

- - - - If 3.a. was “intrauterine antibiotic treatment”:
        - Which antibiotic drug do you use most of the times? (SC)

Amoxicillin pills

Other:

Comment box

- - - - If 3.a. was “intrauterine antibiotic treatment” or “systemic treatment:
        - On which criterion is your usual treatment duration depending? (SC)

Infectious agent

Clinical signs

Degree of infection according to microbiology

Other:

Comment box

- 1. Do you perform uterine lavages routinely in CIE cases? (SC)
     - Yes
     - No
     - No, only exceptionally
       - If 3.b. was “Yes” or “No, only exceptionally”:
         - What do you use for uterine lavages? (MC)

0.9 % saline

Tap water

Ethacridin lactate solution

Diluted iodine solution

DMSO

Diluted chlorhexidine solution

N-acetylcysteine

Coca Cola

Kerosene

Other:

Comment box

- 1. Do you use oxytocin for CIE treatment? (SC)
     - Yes, routinely
     - No, never
     - Only when presence of echogenic was detected ultrasonographically
     - After every uterine lavage
  2. Do you perform vulvoplasty surgery in cases of poor vulvar closure? (SC)
     - Always
     - Often
     - Only if clinical signs are present
     - Rarely
     - Never
  3. Do you perform control sampling after completion of CIE treatment? (SC)
     - Yes
     - Yes, mostly
     - No, only exceptionally
     - No
  4. According to your experience, how many mares get pregnant after treatment of CIE? (SC)
     - Per cycle:
       - Less than 10 %
       - Less than 30 %
       - Less than 50 %
       - Less than 70 %
       - Less than 90 %
     - Per season:
       - Less than 10 %
       - Less than 30 %
       - Less than 50 %
       - Less than 70 %
       - Less than 90 %

1. Management of PBIE
   1. According to your experience, which mares are susceptible to PBIE? (MC)
      - Young maiden mares (3 to 4 years old)
      - Older maiden mares (older than 4 years of age)
      - Foaling mares
      - Barren mares
      - Slipped mares
      - Mares with a history of abortion/resorption
      - Mares with a history of PBIE
      - Older mares with one or more foals
      - Other:
        - Comment box
   2. How long after detection of ovulation do you perform uterine lavages? (SC)
      - Until 1 day after ovulation (post ov.)
      - Until 2 days post ov.
      - Until 3 days post ov.
      - Until 4 days post ov.
      - Until 5 days post ov.
      - Until 6 days post ov.
      - Longer
   3. According to your experience, how many mares get pregnant after treatment of PBIE? (SC)
      - Per cycle:
        - Less than 10 %
        - Less than 30 %
        - Less than 50 %
        - Less than 70 %
        - Less than 90 %
      - Per season:
        - Less than 10 %
        - Less than 30 %
        - Less than 50 %
        - Less than 70 %
        - Less than 90 %
2. Abandoned diagnostic and treatment procedures
   1. Which diagnostic methods for endometritis did you give up?
      - Comment box
   2. Which treatment methods for endometritis did you give up?
      - Comment box
